# Construction of a CXC Chemokine-Based Prediction Model for the Prognosis of Colon Cancer

**DOI:** 10.1155/2020/6107865

**Published:** 2020-03-30

**Authors:** Kaisheng Liu, Minshan Lai, Shaoxiang Wang, Kai Zheng, Shouxia Xie, Xiao Wang

**Affiliations:** ^1^The First Affiliated Hospital of Southern University of Science and Technology, The Second Clinical Medical College of Jinan University, Shenzhen People's Hospital, Shenzhen, Guangdong, China; ^2^School of Pharmaceutical Sciences, Shenzhen University Health Science Center, Shenzhen, Guangdong, China; ^3^Zhejiang Province Key Laboratory of Anti-Cancer Drug Research, College of Pharmaceutical Sciences, Zhejiang University, Hangzhou, China

## Abstract

Colon cancer is the third most common cancer, with a high incidence and mortality. Construction of a specific and sensitive prediction model for prognosis is urgently needed. In this study, profiles of patients with colon cancer with clinical and gene expression data were downloaded from Gene Expression Omnibus and The Cancer Genome Atlas (TCGA). CXC chemokines in patients with colon cancer were investigated by differential expression gene analysis, overall survival analysis, receiver operating characteristic analysis, gene set enrichment analysis (GSEA), and weighted gene coexpression network analysis. CXCL1, CXCL2, CXCL3, and CXCL11 were upregulated in patients with colon cancer and significantly correlated with prognosis. The area under curve (AUC) of the multigene forecast model of CXCL1, CXCL11, CXCL2, and CXCL3 was 0.705 in the GSE41258 dataset and 0.624 in TCGA. The prediction model was constructed using the risk score of the multigene model and three clinicopathological risk factors and exhibited 92.6% and 91.8% accuracy in predicting 3-year and 5-year overall survival of patients with colon cancer, respectively. In addition, by GSEA, expression of CXCL1, CXCL11, CXCL2, and CXCL3 was correlated with several signaling pathways, including NOD-like receptor, oxidative phosphorylation, mTORC1, interferon-gamma response, and IL6/JAK/STAT3 pathways. Patients with colon cancer will benefit from this prediction model for prognosis, and this will pave the way to improve the survival rate and optimize treatment for colon cancer.

## 1. Introduction

Colon cancer is one of the most common tumors observed in the world [1]. In the United States, colon cancer is the third most commonly diagnosed cancer, and the second most common cause of cancer-related death [2]. In China, colon cancer is the fifth most common cause of cancer-related death [3]. As a result of improvements in treatment and earlier detection, from the mid-1970s to the most recent time period (2006-2012), the 5-year relative survival rate for all stages of colon cancer increased from 51% to 66% [2]. Despite dramatic reductions in colorectal cancer incidence and mortality, striking disparities by age, race, and tumor subsite remain [2, 4]. Colorectal cancer incidence rates are about threefold higher in transitioned versus transitioning countries [4]. Novel biomarkers with clinical value are thus essential to improve compliance rates and predict poor prognoses for colon cancer.

CXC chemokines (CXCLs 1–16) are heparin-binding proteins that display disparate roles in the regulation of angiogenesis, angiostasis, and metastasis in cancer [5]. CXCLs are widely expressed in gastrointestinal cancers and are correlated with prognosis [6–8]. Recently, CXCLs have emerged as putative plasma biomarkers for pancreatic cancer diagnosis [9, 10]. Overexpression of CXCL1 is associated with tumor progression and poor prognosis in hepatocellular carcinoma [11]. CXCL4 is a predictor of tumor angiogenic activity and a prognostic biomarker in patients with non-small-cell lung cancer (NSCLC) undergoing surgical treatment [12]. CXCL5 favors tumor progression by attracting neutrophils [13]. CXCL12 is associated with gallbladder carcinoma progression [14]. Highly expressed CXCL16 is associated with good prognosis and increases tumor-infiltrating lymphocytes in colon cancer [15]. In this study, we investigated the potential of CXCLs as prognostic biomarkers for colon cancer.

This study is the first to report that the prediction model based on the risk score of the multigene model and three clinicopathological risk factors can predict the survival of patients with colon cancer, indicating that patients with colon cancer will benefit from this prediction model to improve survival rate.

## 2. Materials and Methods

### 2.1. Patient Data

Profiles of patients with colon cancer were downloaded from the GSE41258, GSE68468, and GSE44076 datasets of Gene Expression Omnibus (GEO) database and The Cancer Genome Atlas (TCGA) database. For expression difference analysis, data from 53 normal and 167 tumor samples from GSE41258, 41 normal and 456 tumor samples from TCGA, 54 normal and 236 tumor samples from GSE68468, and 98 normal and 98 tumor samples from GSE44076 were used. The survival data of all patients with tumor samples in GSE41258 and 428 of 456 patients with tumor samples in TCGA were included in the other analyses. GSE68468 and GSE44076 have no survival data and were used solely for differential expression gene analysis. The associations of overall survival and clinic pathological information of the patients were analyzed by univariate and multivariate Cox regression analyses. Correlations between the expression of CXCLs and clinical characteristics of patients with colon cancer were investigated using Pearson's correlation coefficient. Statistics were performed using IBM SPSS Statistics for Windows, version 23.0 (IBM Corp., Armonk, N.Y., USA).

### 2.2. Differential Expression Gene Analysis

Differential expression gene analysis was performed to estimate the difference in gene expression between tumor samples and healthy controls using the “limma” and “edgeR” packages for GEO and TCGA data, respectively, using R (R Core Team, Vienna, Austria) [16, 17]. Consequently, log_2_foldchange (logFC), *P* value, and the false discovery rate (FDR) (or adjusted *P* value) of each gene were obtained. Expression patterns of each CXC chemokine were illustrated by heat map. CXCLs with ∣logFC∣ > 1, *P* value < 0.05, and FDR < .05 were considered as differentially expressed genes (DEGs). A Venn diagram was drawn to show overlapping DEGs from the four datasets. The expression differences of each overlapping DEG were presented in boxplots.

### 2.3. Survival Analysis

Hazard ratios (HRs) and *P* values of overlapping DEGs were calculated by univariate Cox analysis in R. Survival analysis of patients in regard to the overlapping DEGs was conducted using the Kaplan-Meier method in R and based on the gene expression in tumor samples and overall survival of the patients. Survival curves were plotted to show the differences in patient survival between high- and low-expression groups. *P* < 0.05 was considered significant.

### 2.4. Forecast Model Construction

The risk scores of each patient were calculated from the expression of DEGs and overall survival using multivariate Cox regression analysis in R. Based on these risk scores, receiver operating characteristic (ROC) curves were plotted to demonstrate effectiveness in predicting patients' overall survival. The area under curve (AUC) value on each curve indicates predictive accuracy, demonstrated by AUC > 0.60. Survival curves showing differences in patients with different risk scores were drawn by dividing the patients into high- and low-risk groups. Risk score distribution figures and survival time figures were also plotted.

### 2.5. Nomogram Construction and Assessment

Nomograms for individualized prediction were generated based on risk scores from the multigene models and clinical risk factors to predict 3-year and 5-year overall survival (OS) using the “rms” package in R. Concordance index (C-index), ROC curve (AUC), and calibration plots were obtained using R to evaluate the performance of the nomograms.

### 2.6. Pathway Analysis

The potential biological pathways of CXCLs were investigated by gene set enrichment analysis (GSEA) [18], a computational method that determines whether an *a priori* defined set of genes shows statistically significant differences between two biological states. Gene sets enriched in low- and high-risk patient groups were obtained using the expression profiles of patients' tumor samples by java GSEA. KEGG gene sets (v6.2), oncogenic signature gene sets (v6.2), and hallmark gene sets (v6.2) were chosen as references in this study. Gene sets whose results are *P* < 0.01 and FDR < 0.25 were considered significant.

### 2.7. Coexpression Network Analysis

Genes coexpressed with CXCLs were screened by performing weighted gene coexpression network analysis (WCGNA) [19], a biological method for describing the correlation patterns among genes across microarray samples. The network was drawn via Cytoscape (v3.6.1).

## 3. Results and Discussion

### 3.1. Clinical Characteristics of Patients with Colon Cancer

Relationships between the clinical characteristics and OS of patients with colon cancer in GSE41258 and TCGA were clarified by performing univariate and multivariate Cox regression analyses. In univariate analysis, poor OS of patients was significantly related to advanced tumor-node-metastasis (TNM) stage, T3 and T4 stages, N2 and N3 stages, and M1 stage in both GSE41258 and TCGA (Tables 1 and 2,). Characteristics with significant *P* values from the univariate analysis were screened using multivariate analysis. Multivariate analysis revealed that N stage and M stage in GSE41258 and T stage and M stage in TCGA might be independent prognostic factors for patients with colon cancer (Tables 1 and 2). Additionally, the correlations between the clinical characteristics of colon cancer and expression of CXCLs were also investigated. The expression of several CXCLs was significantly related to TNM stage, N stage, M stage, and p53 mutants in GSE41258 (Table 3) and associated with age, TNM stage, N stage, and M stage in TCGA (Table 4).

### 3.2. Identification of CXCLs Differentially Expressed between Tumor and Normal Samples

**Figure 1:**
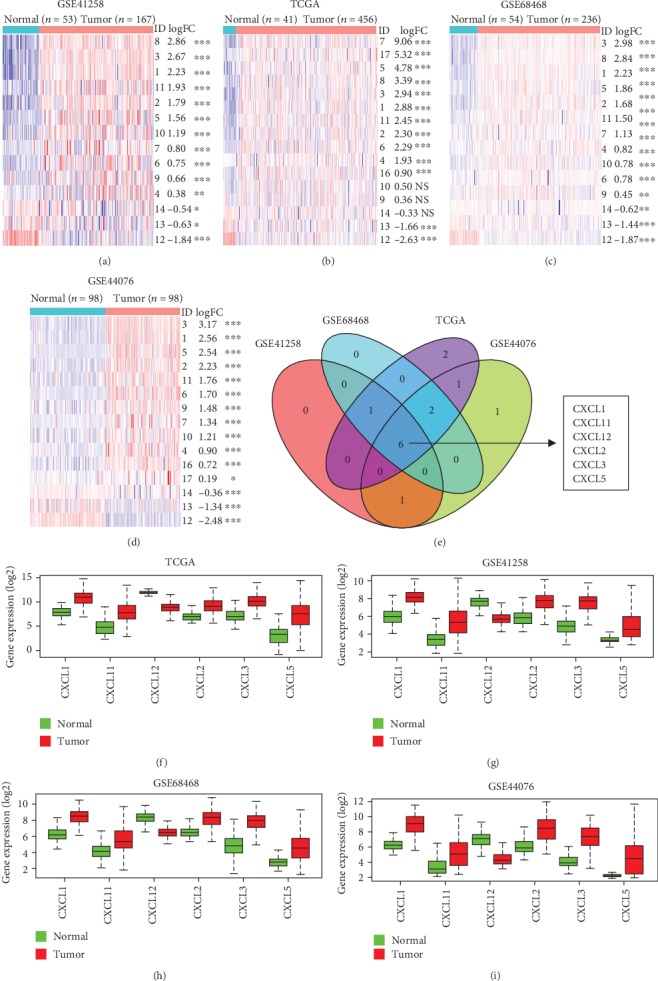
Aberrant expression of CXCLs (CXCLs) in colon cancer. (a–d) Heat maps showing the expression differences in CXCLs between tumor and normal samples in the order of descending logFC based on GSE41258, TCGA, GSE68468, and GSE44076 datasets. The blue and red colors represent low and high expression, respectively. ^∗∗∗^*P* < 0.001; ^∗∗^*P* < 0.01; ^∗^*P* < 0.05; ^NS^*P* > 0.05. CXCLs with *P* < 0.05, FDR < 0.05, and ∣logFC∣ > 1 were identified as DEGs. (e) Venn diagram displaying the overlapping DEGs in the aforementioned datasets, including CXCL1, CXCL11, CXCL12, CXCL2, CXCL3, and CXCL5. (f–i) Boxplots representing the different expression levels of the overlapping genes in tumor and normal samples according to TCGA, GSE41258, GSE68468, and GSE44076 datasets.

To systematically identify CXC chemokine DEGs in colon cancer, we compared their expression levels between tumor and normal samples. In the GSE41258, TCGA, GSE68468, and GSE44076 datasets, 8/14, 12/16, 9/14, and 11/15 CXC chemokine genes, respectively, were found significantly aberrantly expressed in colon cancer (Figures 1(a)–1(d)). Furthermore, the Venn diagram demonstrated that a total of six DEGs, including CXCL1, CXCL11, CXCL12, CXCL2, CXCL3, and CXCL5, overlapped in the aforementioned datasets (Figure 1(e)). Among these, CXCL1, CXCL11, CXCL2, CXCL3, and CXCL5 were all upregulated, whereas CXCL12 was downregulated in tumor samples compared to normal tissue. The expression differences between tumor and normal tissues from each dataset are shown by boxplot (Figures 1(f)–1(i)). Expression difference analysis revealed that many CXCLs, especially the overlapping DEGs (CXCL1, CXCL11, CXCL12, CXCL2, CXCL3, and CXCL5), have the potential to be promising diagnostic biomarkers for colon cancer.

We also analyzed the effects of the expression of the overlapping DEGs on patients' survival by univariate Cox analysis and the Kaplan-Meier method in patients with colon cancer. In univariate Cox analysis and overall survival curves, expression of CXCL11, CXCL2, and CXCL3 in GSE41258 and CXCL1, CXCL2, and CXCL3 in TCGA had a strong correlation with the progression of colon cancer ([Supplementary-material supplementary-material-1]).

### 3.3. Assessment of the Prognostic Values of CXCL1, CXCL11, CXCL2, and CXCL3 for Patients with Colon Cancer

**Figure 2:**
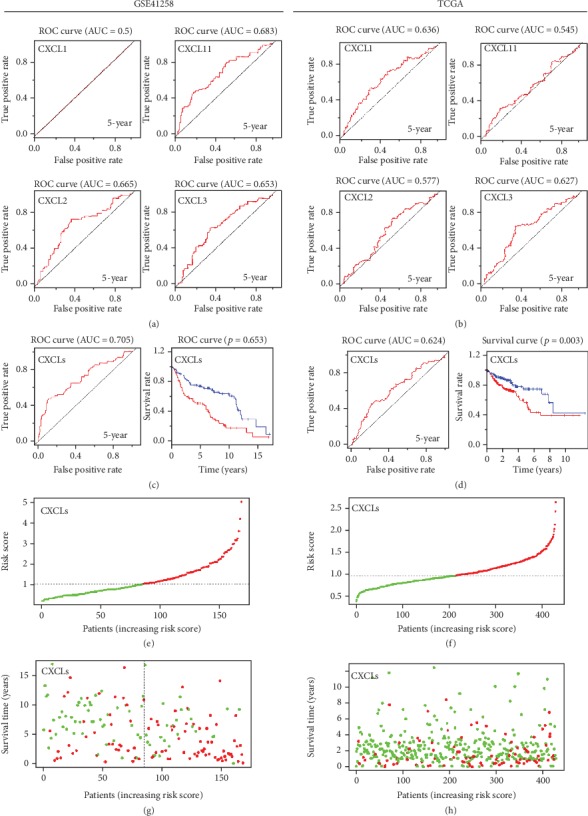
Forecast models predicting the prognosis of patients with colon cancer. (a, b) Single-gene models of CXCL1, CXCL2, CXCL3, and CXCL11 in GSE41258 and TCGA. (c, d) Multigene forecast models based on the expression of CXCL1, CXCL2, CXCL3, and CXCL11, collectively. ROC curves and survival curves of the multigene forecast models in GSE41258 and TCGA, respectively. (e, f) Risk score distribution of patients according to the multigene forecast model in GSE41258 and TCGA. The green dots and red dots represent low-risk and high-risk, respectively. (g, h) Survival times and statuses of patients according to the multigene forecast model in GSE41258 and TCGA. The green dots and red dots represent alive and dead status, respectively.

To evaluate the prognostic values of CXCL1, CXCL11, CXCL2, and CXCL3, we further constructed forecast models by plotting ROC curves based on multivariate Cox regression analysis. Results showed that single-gene models of CXCL11, CXCL2, and CXCL3 in GSE41258 and single-gene models of CXCL1 and CXCL3 in TCGA exhibited the potential ability to predict 5-year OS for patients with colon cancer (AUC > 0.60) (Figures 2(a) and 2(b)). ROC curves of each gene for 3-year OS are shown in [Supplementary-material supplementary-material-1]. To assess the joint effects of CXCL1, CXCL11, CXCL2, and CXCL3 on patients' survival, a multigene forecast model was established. Using R package, risk scores of patients were calculated according to the below formulas: risk score (GSE41258) = (0.486∗CXCL1_Exp_) + (−0.278∗CXCL11_Exp_) + (−0.727∗CXCL2_Exp_) + (0.128∗CXCL3_Exp_) and risk score (TCGA) = (−0.124∗CXCL1_Exp_) + (−0.063∗CXCL11_Exp_) + (−0.038∗CXCL2_Exp_) + (0.006∗CXCL3_Exp_). As a result, AUCs from the multigene forecast model in GSE41258 and TCGA were both >0.60 (0.705 in GSE41258 and 0.624 in TCGA) (Figures 2(c) and 2(d)). ROC curves of multigene analysis for 3-year OS are shown in [Supplementary-material supplementary-material-1]. These results suggest that the forecast model possessed moderate specificity and sensitivity in colon cancer survival prediction. Further, according to the median risk score, patients were divided into low-risk and high-risk groups and survival curves were plotted. Low-risk patients had better survival than that of the high-risk group (*P* < 0.001 in GSE41258, *P* = 0.003 in TCGA; Figures 2(c) and 2(d)). The risk score distribution of patients in the order of ascending risk score is presented (Figures 2(e) and 2(f)). Survival times and status figures showed that the number of deceased patients in the high-risk group was higher than that in the low-risk group (Figures 2(g) and 2(h)), which was reflected by the survival curves. Collectively, these findings showed that the forecast model based on the expression of CXCL1, CXCL11, CXCL2, and CXCL3 could have a high prognostic value for the survival of patients with colon cancer.

### 3.4. Construction of Nomograms Based on the Risk Scores of Multigene Models and Clinical Risk Factors

**Figure 3:**
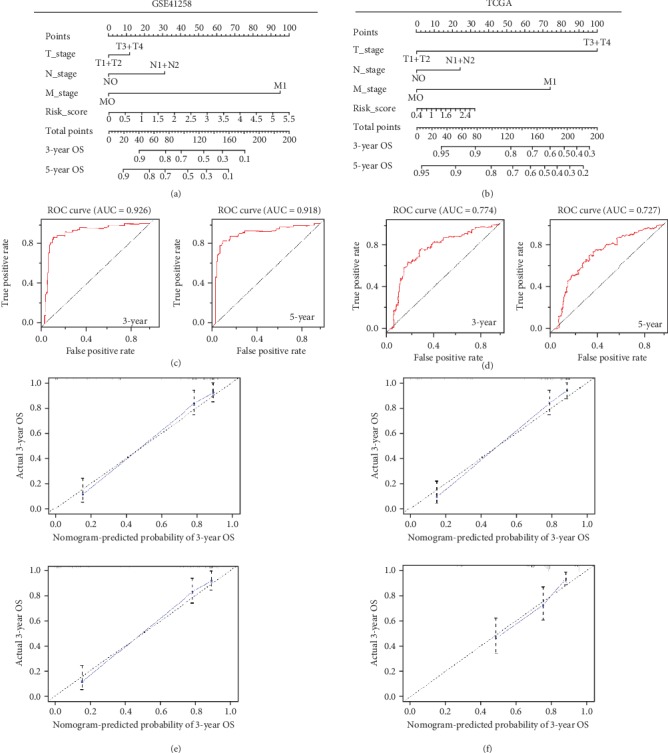
Nomograms predicting 3-year and 5-year OS for patients with colon cancer. (a, b) Nomograms that integrate the risk scores of multigene models and three clinical risk factors (T stage, N stage, and M stage) in GSE41258 and TCGA. (c, d) ROC curves of nomograms in GSE41258 and TCGA. (e, f) Calibration curves for nomograms in GSE41258 and TCGA.

For a more sensitive predictive tool in clinical practice, we constructed nomograms integrating the risk scores of multigene models and three clinicopathological risk factors (T stage, N stage, and M stage) (Figures 3(a) and 3(b)). The *C*-indices of nomograms from GSE41258 and TCGA were 0.812 and 0.737, respectively. For GSE41258, the 3-year and 5-year true positive rates of the nomogram could reach up to 92.6% and 91.8%, respectively (Figure 3(c)), demonstrating that the nomogram was highly accurate in predicting individual OS for colon cancer. The 3-year and 5-year AUCs of the nomogram for TCGA were 0.774 and 0.727, respectively (Figure 3(d)), indicating that this nomogram possesses moderate predictive accuracy for patients' OS. Additionally, the calibration curves for predicting 3-year and 5-year OS also indicated that the nomogram-predicted survival closely corresponded with actual survival outcomes in both GSE41258 and TCGA (Figures 3(e) and 3(f)).

### 3.5. Mechanism of the Effect of CXCL1, CXCL11, CXCL2, and CXCL3 on Colon Cancer Progression

**Figure 4:**
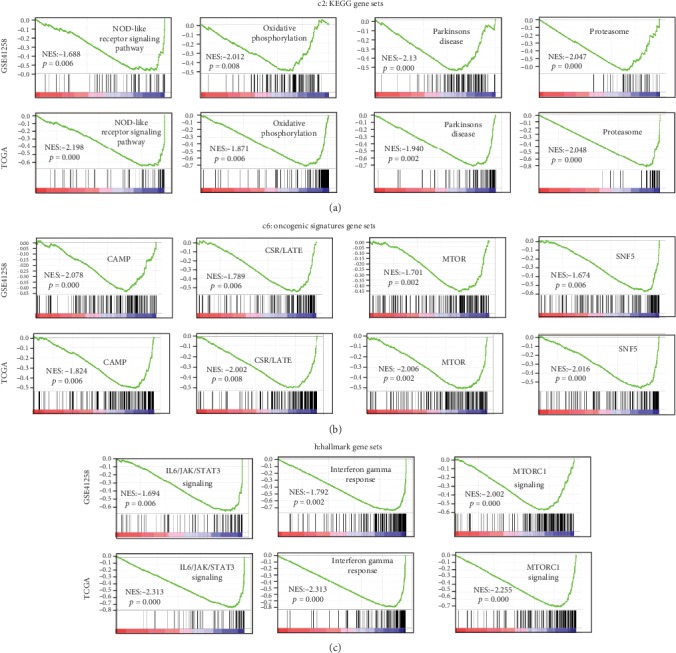
GSEA results based on the risk scores of the multigene forecast model. (a) Significantly enriched gene sets using KEGG gene sets (*c2*) as reference. (b) Significantly enriched gene sets according to oncogenic signature gene sets (*c6*). (c) Significantly enriched gene sets based on hallmark gene sets (*h*).

**Figure 5:**
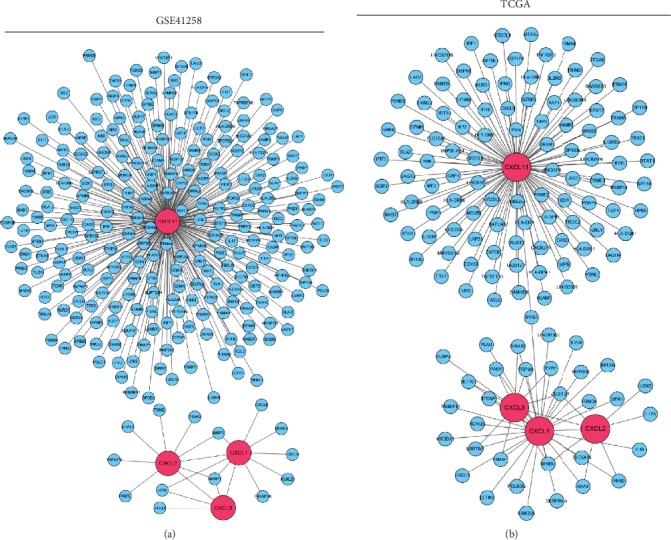
Coexpression network of CXCLs. (a, b) Visualization networks of the genes coexpressed with CXCLs in GSE41258 and TCGA. The blue nodes are the coexpressed genes. The pink nodes are CXCLs.

To identify the mechanism of the effect of CXCL1, CXCL11, CXCL2, and CXCL3 on colon cancer, we performed GSEA and WGCNA. For GSEA, the expression profiles of tumor samples were divided into the low-risk and high-risk groups based on the risk scores of the multigene forecast. Then, the expression profile was analyzed using KEGG gene sets (*c2*), oncogenic signatures gene sets (*c6*), and Hallmark gene sets (*h*) as references. The gene sets of NOD-like receptor signaling pathways, oxidative phosphorylation, and Parkinson's disease and the proteasome were significantly enriched according to *c2* (Figure 4(a)). Based on *c6*, the enriched gene sets were *CAMP*, *CSR/LATE*, *MTOR*, and *SNF5* (Figure 4(b)). Using *h* for reference, mTORC1 signaling, interferon-gamma response, and IL6/JAK/STAT3 signaling were significantly enriched (Figure 4(c)). For WGCNA, coexpressed genes with weights > 0.4 were selected and shown in visualized networks (Figures 5(a) and 5(b)). These results indicate that CXCLs play important roles in the progression of colon cancer.

## 4. Conclusions

Colon cancer is one of the most common and aggressive human malignancies [20, 21]. Despite advances in systemic therapy for colon cancer, successful therapeutic strategies are limited because of the poor prognosis and high recurrence rate [22, 23]. In this study, we constructed a prediction model for the prognosis of patients with colon cancer. In addition, we analyzed the underlying mechanisms of CXCLs by GSEA and built a regulatory network of these chemokines in colon cancer progression.

A few genes were identified to predict the diagnosis and prognosis of colorectal cancer, and the regulatory network was constructed [24–26]. In addition, the DNA methylation was analyzed in colon cancer, and several genes were identified [27]. In this study, we applied a bioinformatics approach to the discovery of prognostic biomarkers in human colon cancer. We assembled gene expression data involving human colon cancers from TCGA and GEO and then searched for differentially expressed genes. Genes associated with patient survival of colon cancer could be identified as single prognostic biomarkers. Using this approach, we identified CXCL1, CXCL11, CXCL2, and CXCL3 as potential biomarkers; we then established a multigene forecast model combining these chemokines. Results showed that our forecast model exhibited the potential ability to predict 5-year OS for patients with colon cancer accurately. We further constructed nomograms integrating the risk scores of multigene models and three clinicopathological risk factors. Results showed that the nomograms have high accuracy in predicting individual OS for colon cancer. We then performed GSEA to find signaling pathways related to CXCLs. This revealed that CXCLs were correlated with the development and progression of tumors. We finally set up a regulatory network of CXCLs in colon cancer. However, the underlying mechanisms need to be further elucidated in future work.

Previous studies indicated that CXCL1 promotes tumor growth and is associated with poor survival in gastric cancer, breast cancer, and hepatocellular carcinoma [11, 28, 29]. However, in the TCGA database, highly expressed CXCL1 is associated with better survival in colon cancer, and this is consistent with a previous report that overexpression of CXCL1 positively correlates with improved survival [30]. CXCL2 is correlated with prognosis in bladder cancer [31]. In our study, CXCL2 was found to be highly expressed and correlated with the survival of patients with colon cancer in GSE41258. CXCL3 plays a predominant role in the tumorigenicity of prostate cancer cells and is upregulated in prostate cancer [32, 33]. It is also involved in the migration, invasion, proliferation, and tubule formation of trophoblasts [34]. CXCL5 is overexpressed in pancreatic cancer, and it is associated with poor survival in hepatocellular carcinoma, pancreatic cancer, and late-stage gastric cancer [35–37]. Interestingly, it has been reported that low expression of CXCL5 is significantly associated with poor prognosis for patients with colorectal cancer [38]. However, CXCL5 had no significant correlation with the survival of patients with colon cancer in TCGA and GSE41258 in our study. CXCL8 has the potential to be a prognostic marker for breast cancer and colorectal cancer [39, 40]. As CXCL8 was not included in GSE44076, it was not referred to in the prediction model in our work. Neuroendocrine-like cell-derived CXCL10 and CXCL11 induce the infiltration of tumor-associated macrophages and lead to the poor prognosis of colorectal cancer [41]. Downregulation of CXCL11 inhibits colorectal cancer cell growth and epithelial-mesenchymal transition [42]. However, highly expressed CXCL11 was found to be related to better survival in GSE41258, but not in TCGA in this study. A high level of CXCL12 is an independent predictor of poor survival in ovarian cancer [43]. Our results showed that CXCL1, CXCL2, CXCL3, and CXCL11 were all upregulated in colon cancer compared with healthy tissues, and in the colon cancer group, a high level of CXCL1, CXCL2, CXCL3, and CXCL11 was correlated with better survival in TCGA or GEO. The differences between this result and previous reports may be due to the differences in patient numbers, age, sex, races, metastasis, complications, or clinical stages.

Using a single gene to predict prognosis is incomplete and limited. Our results indicate that a prediction model using multiple genes and clinical risk factors successfully predicts the prognosis of patients with colon cancer. Patients with colon cancer will benefit from this prediction model to improve treatment options and prognosis.

## Figures and Tables

**Table 1:** Univariate and multivariate Cox regression analyses of overall survival in patients with colon cancer in GSE41258.

Variables	Total *n* = 167*n* (%)	Univariate analysis		Multivariate analysis	
		HR (95% CI)	*P*	HR (95% CI)	*P*
Age					
<60	54 (32.3%)	1 (reference)			
≥60	113 (67.7%)	1.239 (0.782–1.963)	0.361		
Sex					
Male	88 (52.7%)	1 (reference)			
Female	79 (47.3%)	0.661 (0.433–1.008)	0.054		
Group stage					
I+II	69 (41.3%)	1 (reference)		1 (reference)	
III+IV	98 (58.7%)	4.006 (2.461–6.523)	0.000	0.679 (0.275–1.679)	0.402
T stage					
T1+T2	33 (19.8%)	1 (reference)		1 (reference)	
T3+T4	134 (80.2%)	2.531 (1.312–4.884)	0.006	1.219 (0.607–2.446)	0.578
N stage					
N0	84 (50.3%)	1 (reference)		1 (reference)	
N1+N2	83 (49.7%)	2.361 (1.550–3.597)	0.000	2.572 (1.292–5.119)	0.007
M stage					
No	114 (68.3%)	1 (reference)		1 (reference)	
Yes	53 (31.7%)	9.878 (6.295–15.500)	0.000	11.195 (5.949–21.070)	0.000
P53 mutant					
Wild type	46 (27.5%)	1 (reference)			
Mutant	83 (49.7%)	1.118 (0.696–1.795)	0.646		
Missing	39 (23.4%)				

Characteristics with significant *P* values after univariate analysis were screened by multivariate analysis. HR: hazard ratio; CI: confidence interval; TNM: tumor-node-metastasis.

**Table 2:** Univariate and multivariate Cox regression analyses of overall survival in patients with colon cancer in TCGA.

Variables	Total *n* = 428 n(%)	Univariate analysis		Multivariate analysis	
		HR (95% CI)	*P*	HR (95% CI)	*P*
Age					
<60	124 (29.0%)	1 (reference)			
≥60	304 (71.0%)	1.224 (0.762–1.966)	.404		
Sex					
Male	230 (53.7%)	1(reference)			
Female	198 (46.3%)	0.830 (0.547–1.259)	0.380		
TNM stage					
I+II	235 (54.9%)	1 (reference)		1 (reference)	
III+IV	182 (42.5%)	3.318 (2.102–5.238)	0.000	3.018 (0.973–9.362)	0.056
Missing	11 (2.6%)				
T stage					
T1+T2	84 (19.6%)	1 (reference)		1 (reference)	
T3+T4	343 (80.1%)	3.741 (1.515–9.241)	0.005	4.555 (1.087–19.083)	0.038
Missing	1 (0.2%)				
N stage					
N0	251 (58.6%)	1 (reference)		1 (reference)	
N1+N2	177 (41.4%)	2.824 (1.841–4.332)	0.000	0.628 (0.239–1.502)	0.345
M stage					
M0	316 (73.8%)	1 (reference)		1 (reference)	
M1	61 (14.3%)	4.933 (3.101–7.848)	0.000	2.652 (1.502–4.685)	0.001
Missing	51 (11.9%)				

Characteristics with significant *P* values after univariate analysis were screened by multivariate analysis. HR: hazard ratio; CI: confidence interval; TNM: tumor-node-metastasis.

**Table 3:** Correlation of CXC chemokine gene expression and clinical characteristics of patients with colon cancer in GSE41258.

Gene	Age ≥ 60	Sex (female)	Group stage (III+IV)	T stage T3+T4	N stage (N1+N2)	M stage (yes)	P53 (mutant)
CXCL1			-0.252^∗∗^^0.001^		-0.184^∗^^0.017^	-0.252^∗∗^^0.001^	
CXCL2			-0.335^∗∗^^0.000^		-0.283^∗∗^^0.000^	-0.297^∗∗^^0.000^	
CXCL3			-0.280^∗∗^^0.000^		-0.197^∗^^0.011^	-0.269^∗∗^^0.000^	
CXCL4	-0.178^∗^^0.021^	0.179^∗^^0.021^					
CXCL5							
CXCL6							
CXCL7							
CXCL8	0.173^∗^^0.025^						-0.189^∗^^0.032^
CXCL9						-0.250^∗∗^^0.001^	
CXCL10						-0.221^∗∗^^0.004^	
CXCL11						-0.284^∗∗^^0.000^	-0.204^∗^^0.020^
CXCL12				0.171^∗^^0.027^			
CXCL13						-0.203^∗∗^^0.008^	
CXCL14							0.181^∗∗^^0.040^

^∗^Correlation with *P* value < 0.05; ^∗∗^Correlation with *P* value < 0.01.

**Table 4:** Correlation of CXC chemokine gene expression and clinical characteristics of patients with colon cancer in TCGA.

Gene	Age ≥ 60	Sex (female)	Group stage (III+IV)	T stage T3+T4	N stage (N1+N2)	M stage (yes)	P53 (mutant)
CXCL1	0.100^∗^^0.038^		-0.126^∗^^0.010^		-0.117^∗^^0.016^		
CXCL2	0.097^∗^^0.044^		-0.115^∗^^0.018^		-0.121^∗^^0.012^		
CXCL3	0.117^∗^^0.015^		-0.141^∗∗^^0.004^		-0.136^∗∗^^0.005^	-0.120^∗^^0.020^	
CXCL4							
CXCL5							
CXCL6							
CXCL7							
CXCL8							
CXCL9	0.099^∗^^0.040^		-0.179^∗∗^^0.000^		-0.145^∗∗^^0.003^	-0.175^∗∗^^0.001^	
CXCL10			-0.154^∗∗^^0.002^		-0.133^∗∗^^0.006^	-0.146^∗∗^^0.004^	
CXCL11			-0.150^∗∗^^0.002^		-0.142^∗∗^^0.003^	-0.125^∗^^0.015^	
CXCL12							
CXCL13							
CXCL14							
CXCL16		0.119^∗^^0.014^					
CXCL17							

^∗^Correlation with *P* value < 0.05; ^∗∗^Correlation with *P* value < 0.01.

## Data Availability

The data used to support the findings of this study are available from the corresponding author upon request.

## Supplementary Materials

Supplementary Materials Figure S1: survival analysis by CXCLs in colon cancer. (a, b) Forest plots showing the association between the expression of CXCL1, CXCL11, CXCL12, CXCL2, and CXCL3 and overall survival of patients via univariate Cox analysis in GSE41258 and TCGA. After univariate Cox analysis, expression of CXCL11, CXCL2, and CXCL3 in GSE41258 and CXCL1, CXCL2, and CXCL3 in TCGA exhibited significant relationships to lower HRs of death (*P* < 0.05), whereas the other overlapping CXCLs showed no statistical significance. (c, d) Kaplan-Meier overall survival curves of CXCL1, CXCL11, CXCL12, CXCL2, CXCL3, and CXCL5 in GSE41258 and TCGA. Overall survival curves showed that high expressions of CXCL11, CXCL2, and CXCL3 in GSE41258 and CXCL1 and CXCL3 in TCGA were significantly associated with better outcomes of patients' survival (*P* < 0.05). Figure S2: ROC curves to predict the 3-year OS for patients with colon cancer. (a, b) ROC curves in GSE41258 and TCGA, respectively.
