# Supplementary figures and images for: Construction of a CXC Chemokine-Based Prediction Model for the Prognosis of Colon Cancer
Source: Biomed Res Int. 2020 Mar 30;2020:6107865. doi: 10.1155/2020/6107865 (PMC7150705; doi:10.1155/2020/6107865)

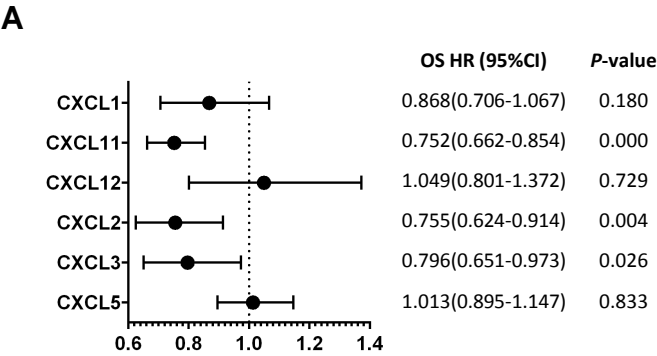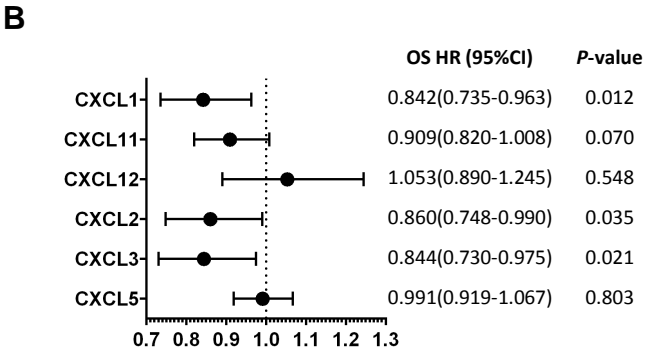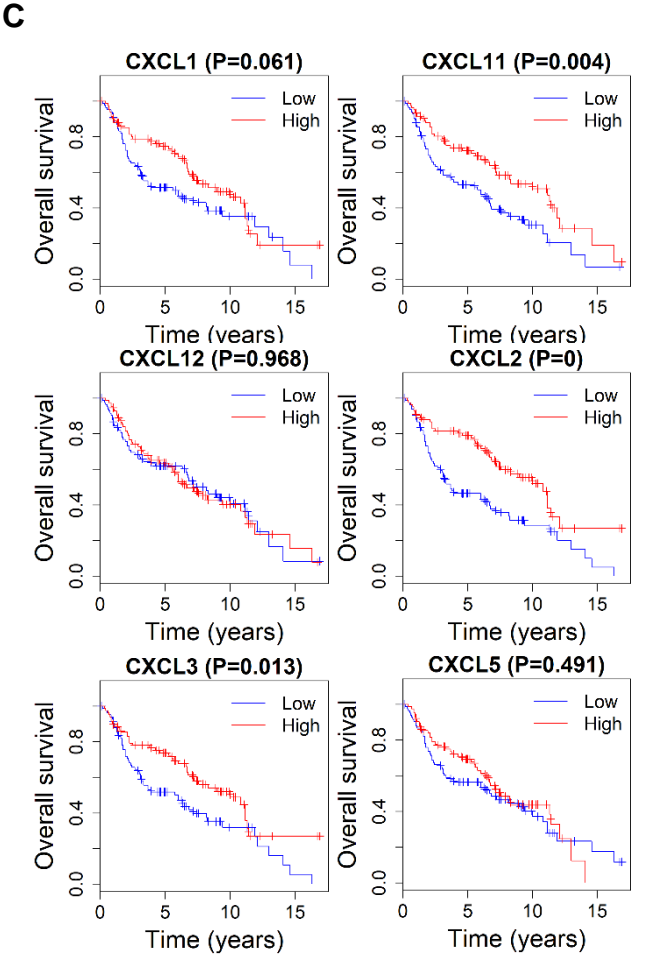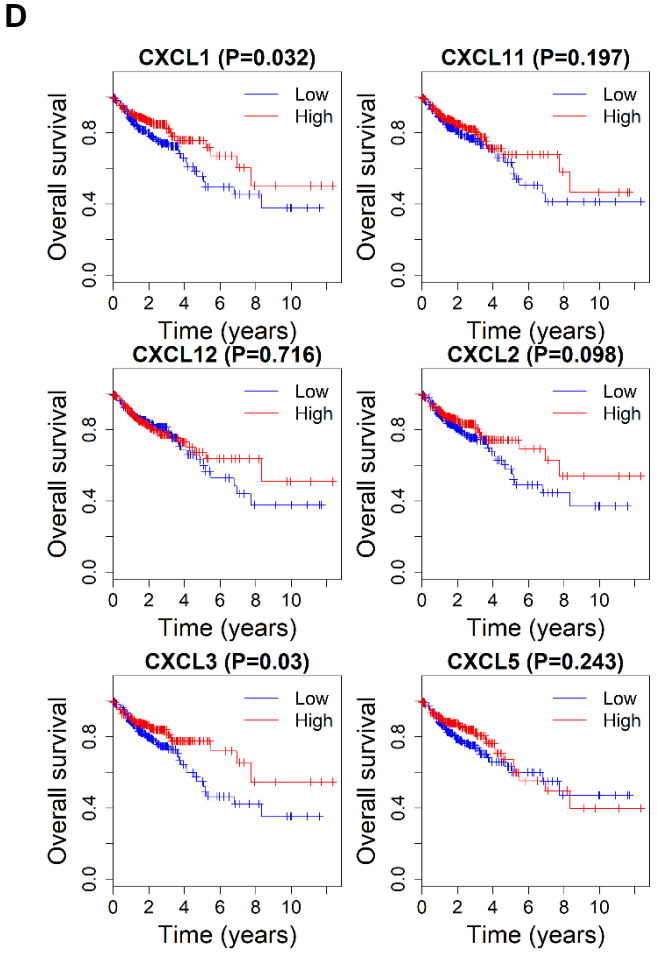

Figure S1

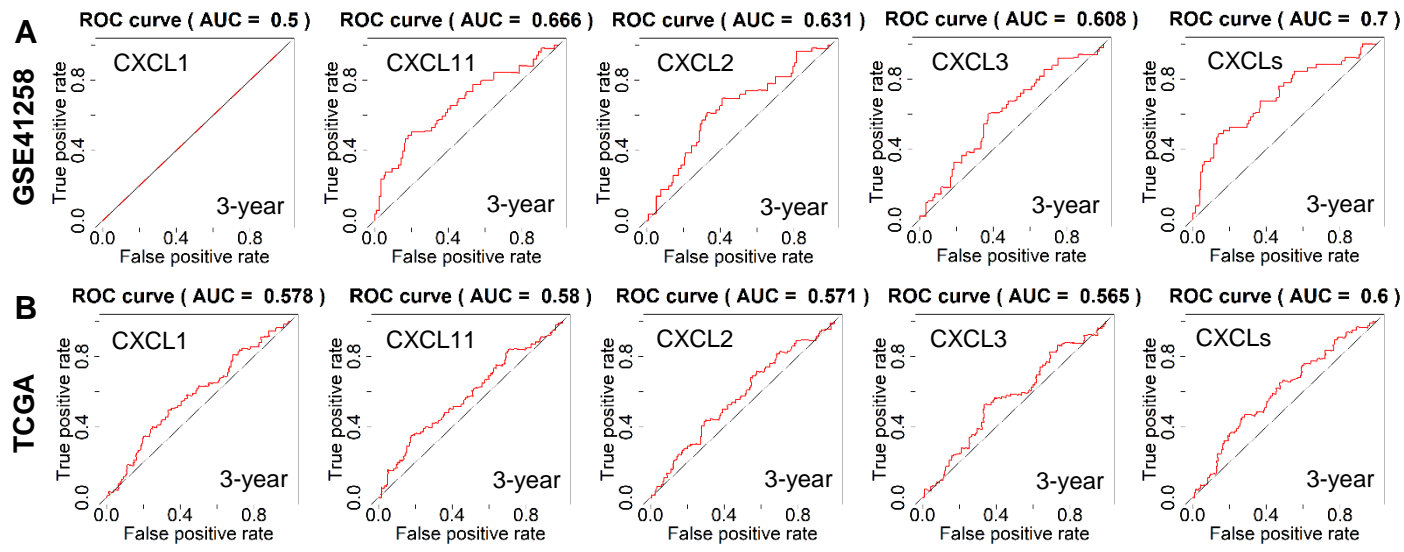

Figure S2

Supplement: Supplementary Materials — Figure S1: survival analysis by CXCLs in colon cancer. (a, b) Forest plots showing the association between the expression of CXCL1, CXCL11, CXCL12, CXCL2, and CXCL3 and overall survival of patients via univariate Cox analysis in GSE41258 and TCGA. After univariate Cox analysis, expression of CXCL11, CXCL2, and CXCL3 in GSE41258 and CXCL1, CXCL2, and CXCL3 in TCGA exhibited significant relationships to lower HRs of death (P < 0.05), whereas the other overlapping CXCLs showed no statistical significance. (c, d) Kaplan-Meier overall survival curves of CXCL1, CXCL11, CXCL12, CXCL2, CXCL3, and CXCL5 in GSE41258 and TCGA. Overall survival curves showed that high expressions of CXCL11, CXCL2, and CXCL3 in GSE41258 and CXCL1 and CXCL3 in TCGA were significantly associated with better outcomes of patients' survival (P < 0.05). Figure S2: ROC curves to predict the 3-year OS for patients with colon cancer. (a, b) ROC curves in GSE41258 and TCGA, respectively. [file 6107865.f1.pdf]
